# Chronic migraine is not associated with cerebellar infarct-like lesions

**DOI:** 10.1186/1129-2377-14-S1-P137

**Published:** 2013-02-21

**Authors:** A Meilan, E Santamarta, A Saiz, D Larrosa, E Cernuda, J Pascual

**Affiliations:** 1 University Hospital Central de Asturias, Spain

## Introduction

Two general population studies [1,2] have found that migraine in general, and especially women with aura, has an increased risk of cerebellar infarct-like lesions by MRI. In addition, there was a trend for higher risk of cerebellar lesions in those migraine subjects with a higher migraine attack frequency, which would have obvious clinical and management implications.

## Objective

To determine whether chronic migraine patients are at increased risk of cerebellar infarct-like lesions on MRI.

## Methods

After signed informed consent, brain MRIs were obtained in 50 women from our headache clinic meeting chronic migraine according to 2006 IHC-II revised criteria. Six had a history of migraine with aura attacks and 19 meet overuse criteria. Their ages ranged from 16 to 63 years (mean 40.9 years) and the length of chronic migraine range from 6 months to 27 years (mean 7.5 years). At least 11 patients had a minimum of one vascular risk factor and the prevalence of right to left shunt with transcranial echo was 58%. Brain MRIs were acquired on a 1.5T unit Signa LX 9.1 (General Electric Systems, USA). Protocol includes whole brain weighted images in saggital T1 (5 mm slices), axial FLAIR T2 (3 mm) and combined proton density and T2 fast spin echo (3 mm). Two independent neuroradiologists carefully analysed all the cerebellar images.

## Results

After an in depth review of all posterior fossa slices, we were unable to find even one cerebellar infarct-like lesion in any of these chronic migraine patients.

## Conclusions

Following the same MRI methodology of the previous studies, we demonstrate that, at least for migrainous women, there is no relationship between migraine frequency and the presence of cerebellar infarct-like lesions. Therefore, at least for the cerebellum, frequency of migraine attacks itself does not seem to be a factor increasing the risk of vascular brain lesions. These findings call for caution when extrapolating findings from the general population into current clinical practice.

Supported by the PI11/00889 FISSS grant (ISCIII)

## References

[B1] Kruit JAMA 2004 14 427 34 10.1001/jama.291.4.427 14747499

[B2] Scher JAMA 2009 14 2563 2570 10.1001/jama.2009.932 19549973 PMC3133433

